# Image-guided microwave ablation of hepatocellular carcinoma (≤5.0 cm): is MR guidance more effective than CT guidance?

**DOI:** 10.1186/s12885-021-08099-7

**Published:** 2021-04-07

**Authors:** Zhaonan Li, Chaoyan Wang, Guangyan Si, Xueliang Zhou, Yahua Li, Jing Li, Dechao Jiao, Xinwei Han

**Affiliations:** 1 Department of Interventional Radiology, The First Affiliated Hospital of Zhengzhou University, No. 1 Jianshe East Road, Zhengzhou City, 450000 Henan Province China; 2 Department of Interventional Radiology, The Affiliated Hospital of Traditional Chinese Medicine of Southwest Medical University, Luzhou, 646000 China

**Keywords:** Hepatocellular carcinoma, Microwave ablation, Interventional radiology, Magnetic resonance imaging

## Abstract

**Background:**

Given their widespread availability and relatively low cost, percutaneous thermal ablation is commonly performed under the guidance of computed tomography (CT) or ultrasound (US). However, such imaging modalities may be restricted due to insufficient image contrast and limited tumor visibility, which results in imperfect intraoperative treatment or an increased risk of damage to critical anatomical structures. Currently, magnetic resonance (MR) guidance has been proven to be a possible solution to overcome the above shortcomings, as it provides more reliable visualization of the target tumor and allows for multiplanar capabilities, making it the modality of choice. Unfortunately, MR-guided ablation is limited to specialized centers, and the cost is relatively high. Is ablation therapy under MR guidance better than that under CT guidance? This study retrospectively compared the efficacy of CT-guided and MR-guided microwave ablation (MWA) for the treatment of hepatocellular carcinoma (HCC ≤ 5.0 cm).

**Methods:**

In this retrospective study, 47 patients and 54 patients received MWA under the guidance of CT and MR, respectively. The inclusion criteria were a single HCC ≤ 5.0 cm or a maximum of three. The local tumor progression (LTP), overall survival (OS), prognostic factors for local progression, and safety of this technique were assessed.

**Results:**

All procedures were technically successful. The complication rates of the two groups were remarkably different with respect to incidences of liver abscess and pleural effusion (*P* < 0.05). The mean LTP was 44.264 months in the CT-guided group versus 47.745 months in the MR-guided group of HCC (*P* = 0.629, log-rank test). The mean OS was 56.772 months in the patients who underwent the CT-guided procedure versus 58.123 months in those who underwent the MR-guided procedure (*P* = 0.630, log-rank test). Multivariate Cox regression analysis further illustrated that tumor diameter (< 3 cm) and the number of lesions (single) were important factors affecting LTP and OS.

**Conclusions:**

Both CT-guided and MR-guided MWA are comparable therapies for the treatment of HCC (< 5 cm), and there was no difference in survival between the two groups. However, MR-guided MWA could reduce the incidence of complications.

## Background

The optimal treatment choice for hepatocellular carcinoma (HCC ≤ 5 cm) is a very complex issue, as the selection of treatment requires careful consideration of multiple factors, including tumor location, liver function, and physical status [1, 2]. The treatments for early HCC include liver transplantation, surgical resection, and local ablation. However, due to the high cost and shortage of donor livers, many patients are not candidates for these radical treatment options. Therefore, most centers regard thermal ablation as the main treatment for early-stage HCC [3–5]. Of note, the European Association for the Study of the Liver (EASL) and the American Association for the Study of Liver Diseases (AASLD) also use local ablation as first-line treatment for patients with early HCC who are not eligible for surgical treatments or as a bridge to transplantation. At present, computed tomography (CT)-guided thermal ablation is generally accepted by most clinical centers [6–9]. However, unenhanced CT cannot clearly demonstrate the boundary of ablated lesions during the ablation process, and multiple uses of contrast agents will undoubtedly increase the burden on the kidneys. Therefore, magnetic resonance imaging (MR) is a promising guidance method for microwave ablation (MWA) that has excellent tissue resolution, does not require ionizing radiation during treatment, and allows for multidirectional imaging.

This study retrospectively analyzed and summarized the results of MWA for HCC (≤5.0 cm) under the guidance of CT and MR, and Cox regression was used to analyze the factors affecting LPT and OS. The results are summarized as follows.

## Methods

### Patients

This was a retrospective cohort study conducted in a single center and approved by the institutional review board. In this retrospective study, we included 47 patients (55.8 ± 8.9 years; range 41–69 years) who received CT-guided MWA for HCC and 54 patients (53.2 ± 6.5, range 43–67 years) who received MWA under MR guidance (median age 56 years; range 36–69 years). The patient characteristics are shown in (Table 1**)**. The inclusion and exclusion criteria are listed in (Table 2**)**.

**Table 1:** Patient characteristics

Characteristics	CT-guided (***n*** = 47)	MR-guided (***n*** = 54)	***P*** value
**Age** (y)†	55.8 ± 8.9	53.2 ± 6.5	0.367^‡^
**Sex**			0.176^*^
Male	31	43	
Female	16	11	
**ECOG performance status**			0.230^*^
0	29	26	
1	18	28	
**Etiology**			0.086^§^
Hepatitis B	32	44	
Hepatitis C	4	6	
Alcohol	9	2	
Unknown	2	2	
**Child–Pugh class**			0.217^*^
A	33	31	
B	14	23	
**Location of tumor**			0.245^*^
Typical locations	23	33	
Hepatic dome	12	7	
Close to the heart/diaphragm/hepatic hilum	12	14	
**a-Fetoprotein level (ng/mL)**			0.407^*^
< 200	28	37	
> 200	19	17	
**Tumor diameter (cm)**			0.163^*^
<3	28	24	
≥ 3,< 5	19	30	
**Tumor number**			0.686^*^
Single (1)	26	33	
2–3	21	21	
**Duration of ablation (min)**	11.2 ± 5.4	9.7 ± 3.2	0.003^‡^
**Generator power (watts)**	57.3 ± 8.5	54.5 ± 6.3	0.047^‡^

Note.—Unless indicated, data are numbers of patients, and numbers in parentheses are percentages. *ECOG* Eastern Cooperative Oncology Group. *Pearson χ2 test was used. †Data are mean ± standard deviation. ‡Independent-samples t test was used. §Fisher exact test was used

**Table 2:** Inclusion and exclusion criteria

Inclusion criteria	***Exclusion criteria***
1 Age range: 18–75 years	Age < 18 or > 75 years
2 HCC diagnosed according to EASL standards	No pathology or evidence on imaging
3 Child–Pugh grade A or B	Child–Pugh grade C
3 BCLC grades are A and B	BCLC grades are C
4 ECOG score ≤ 2	ECOG score > 2
4 Liver lesion number ≤ 3	Liver lesion number > 3
5 Single tumor diameter < 5 cm	Single tumor diameter ≥ 5
6 Expected survival time > 3 months	Expected survival time ≤ 3 months
7 No portal vein thrombus	Portal vein thrombus
8 No extrahepatic metastases	Extrahepatic metastases
9 PLT > 40 × 109/L or PT ≤25 s	PLT ≤ 40 × 109/L or PT > 25 s

European Association for the Study of the Liver, *EASL* Eastern Cooperative Oncology Group, *ECOG* platelet, *PLT* prothrombin time, *PT*; *HCC* hepatocellular carcinoma

### Preparation before treatment

All procedures were performed by an alternating team of two trained interventional radiologists with 8–10 years of experience in ablation procedures. The patient’s positioning was determined according to the preoperative puncture plan on CT/MR. All treatment procedures were performed under local anesthesia.

### CT-guided ablation procedure protocol

**Fig. 1:**
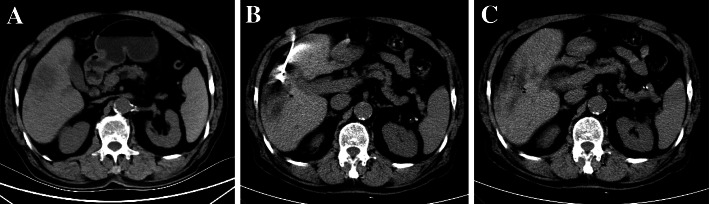
CT imaging before ablation therapy **a** and after placement of an MWA probe **b**. Postinterventional CT imaging after removal of the MWA probe shows a non-enhancing ablation zone **c**. The path of the former probe track can still be seen lateral to the ablation zone. In the center of the ablation zone the formation of bubbles after tissue ablation

Under the guidance of CT, the predesigned procedure for lesion site puncture was conducted. The microwave ablation probe (ECO-100AI10, ECO Microwave System Co, Nanjing, China) was inserted after achieving the best insertion angle and depth, and 57.3 ± 8.5 (watts) of ablation was used. The time settings were typically 11.2 ± 5.4 min. After ablation was complete, enhanced CT was immediately used to assess the ablation area, to provide supplementary treatment for the residual tumor after ablation and to evaluate immediate complications **(**Fig. 1**)**.

### MR-guided ablation procedure protocol

**Fig. 2:**
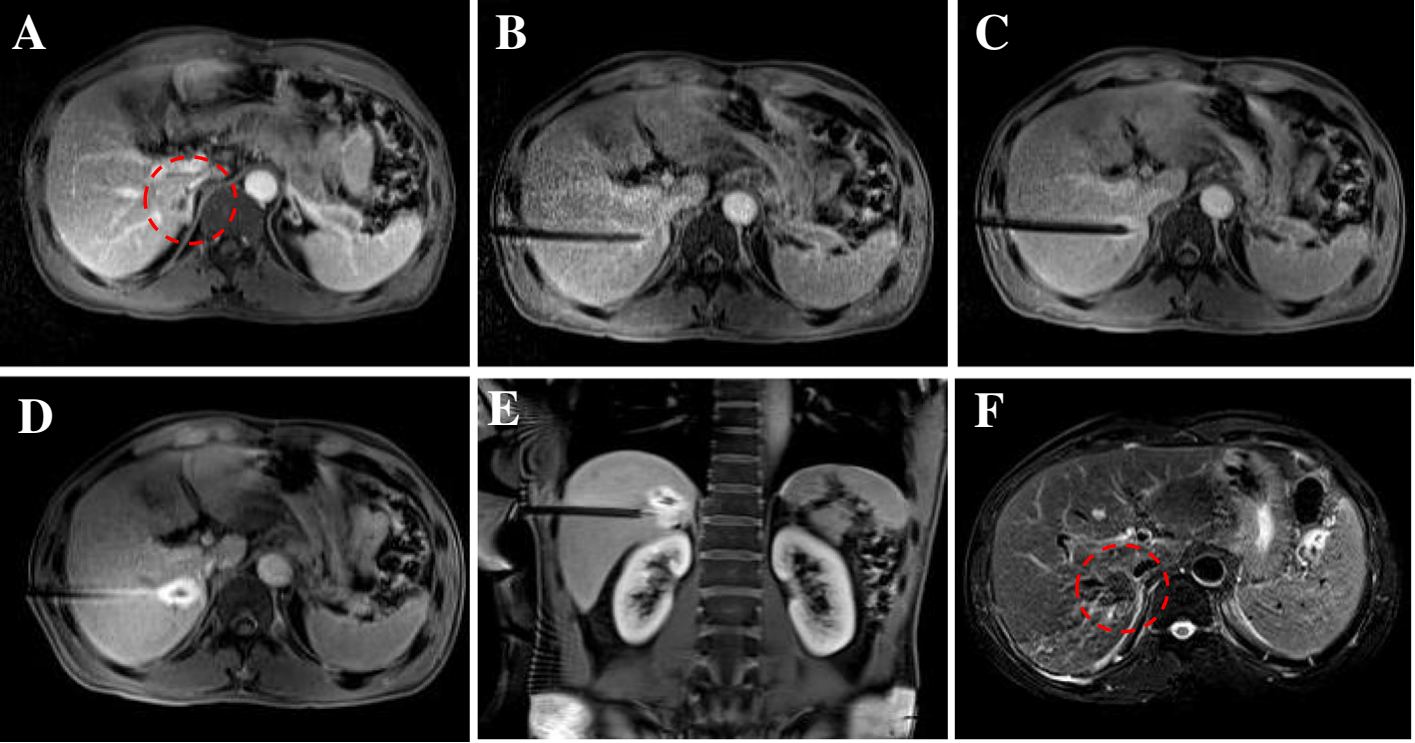
Images of a 58-year-old patient with a small HCC (8.8 mm) in the caudate lobe (**a**; T1WI dashed circle). First, with the guidance of MR, the probe was accurately inserted into the target lesion to finish the ablation procedure (**b, c**; T1WI). After the treatment, the thermal-induced damage zone estimated as hyperintensity on the T1 high signal range completely covers the tumor after ablation **d**. Then, a typical “target sign” is clearly shown in the ablated area of T1WI **d**, **e**, and a low point appears in T2WI immediately after MWA **f**; dashed circle)

**Fig. 3:**
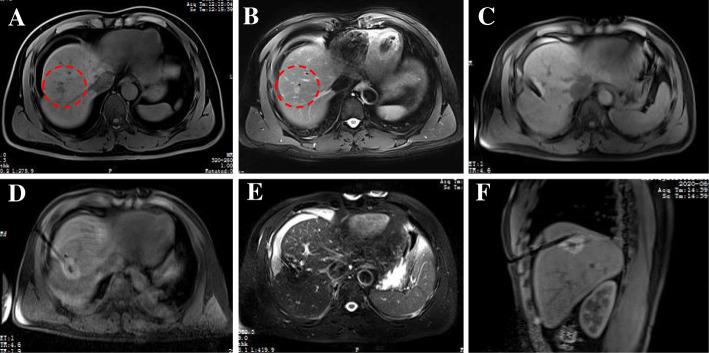
Small HCC in the liver of a 48-year-old man treated with MR-guided MWA. Nodules with a diameter of 12 mm are located in the right lobe of the liver. Before MWA, the nodules have a low signal on T1WI (**a**; dashed circle) and appear with hyperintensity on T2WI (**b**; dashed circle). Then, MR was used to proform the puncture path in the T1WI sequence and reconfirm it through the T1WI traverse after reaching the tumor target **c**. After MWA, a typical “target sign” is clearly shown in the ablated area of T1WI **d**, **f**, and a low point appears in T2WI immediately after MWA **e**

For this study, an MR-compatible MWA apparatus (2450 MHz, ECO Medical Instrument Co., Ltd. Nanjing, China) was used, and the MWA procedure was guided by a 3.0 T dual gradient MR scanner (Magnetom Verio 3.0 T scanner, Siemens Healthineers, Germany) with a closed bore (inner diameter, 70 cm). After marking with the cod liver oil capsule matrix on the body surface, a standard MR protocol was carried out to locate intrahepatic lesions. Under the guidance of MR, a microwave probe (ECO-100AI13, 1.8 mm, 15 cm, Co., Ltd. Nanjing, China) was inserted into the tumor center, and multiple scans were carried out to confirm that the applicator tip was beyond the distal tumor 0.5–1 cm. Additionally, tumors at each site were ablated at 54.5 ± 6.3 watts for 9.7 ± 3.2 min **(**Figs. 2 and 3**)**. During ablation, a series of monitoring T2 Haste and T1 Vibe sequences were performed continuously to monitor the ablated scope every 16 s. If MR imaging showed that the ablation area did not cover 110% of the lesion, the probe was requisitioned, and multiple overlapping ablations were needed. The MR scanning sequences and parameters used in our study are listed in (Table 3**)**.

**Table 3:** MR scanning sequences and parameters

Section	***Sequence***	***TE (ms)***	***TR (ms)***	***Slice thickness (mm)***	***Matrix***	***Flip angle***	***Band Width (Hz/pixel)***
**Transverse section**	T1 Vibe	1.93	4.56	3.3	216 × 288	9.0	400
**Transverse section**	T2 Haste	106	1000	4.5	137 × 256	180	781
**Transverse section**	Diffusion	83	7100	5.0	192 × 144	90	1670
**Coronal section**	T1 vibe	2.46	6.11	3.0	179 × 256	9.0	410
**Sagittal**	T2 Haste	106	1000	4.0	137 × 256	180	781

### Definitions and evaluation of data

The study endpoints were OS, LTP, and radiological response. OS was defined as the interval between initial treatment and death from any cause. LTP was defined as the detection of nodular enhancement in the adjacent ablation area based on follow-up imaging data. Radiological response was evaluated according to the modified response evaluation criteria in solid tumors (mRECIST; 2020 edition) 4 weeks after MWA.

### Follow-up

One month after MWA, laboratory tests including tumor markers (i.e., alpha-fetoprotein (AFP)) and liver function tests, as well as imaging studies (i.e., enhanced CT or enhanced MR), were performed. Subsequently, the patients were followed-up every 3 months to monitor recurrence/residual disease after MWA. Treatment response was evaluated using the mRECIST (2020 edition). When the patient did not achieve complete response (CR), additional treatment was performed at the physicians’ discretion until CR was achieved.

### Statistical analysis

Statistical analysis was conducted using the statistical software SPSS 22.0 (SPSS Inc., Chicago, IL, USA). To determine significant differences between the two groups, continuity correction and independent-samples t-test, Pearson χ2 test, and Fisher exact test were used. Categorical variables are expressed as numbers or percentages (%), and continuous variables are expressed as the mean ± standard deviation (SD). Comparisons between two groups were conducted using a chi-square test or Wilcoxon rank-sum test. Kaplan–Meier survival curves were used for survival analysis. Univariate and multivariate Cox proportional hazards regression analyses were used to predict prognostic factors of LTP and OS. *P* < 0.05 was considered to indicate significant differences.

## Results

### Patient characteristics

**Fig. 4:**
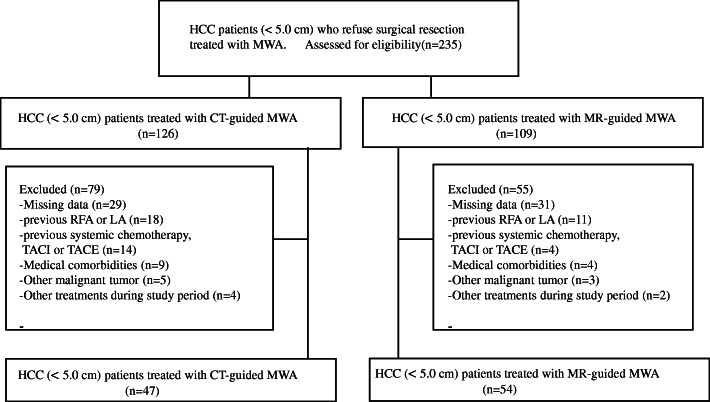
Flow diagram showing the exclusion criteria. RFA = radiofrequency ablation; LR = liver resection; LT = liver transplantation; TACI = transarterial chemoinfusion; TACE = transarterial chemoembolization

A total of 235 patients with HCC underwent CT-guided MWA or MR-guided MWA during the study period. A total of 134 patients were excluded from the study based on the exclusion criteria. Thus, 101 patients with HCC ≤ 5.0 cm were ultimately included in the present study (CT-guided, *n* = 47; MR-guided, *n* = 54) **(**Fig. 4**)**. There were no significant differences in terms of age, sex, ECOG score, etiology, Child–Pugh classification, tumor location, or tumor diameter between the two groups. For patients in the CT-guided group and in the MR-guided group, the mean energy of each tumor was 57.3 ± 8.5 watts and 54.5 ± 6.3 watts, respectively, and the difference between the two groups was statistically significant (*P* = 0.047). Additionally, the mean ablation duration of each tumor was 11.2 ± 5.4 and 9.7 ± 3.2, for the CT-guided group and MR-guided group, respectively (*P* = 0.003).

### Safety of CT guidance versus safety of MR guidance

Postoperative pain and fever (with/without treatment) were the most common adverse events after treatment. With four exceptions, all adverse events and complications were CTCAE grade 1 or 2 (mild symptoms, no or local/noninvasive intervention indicated) or interventional radiology society grade A or B (no or nominal treatment, no consequences). Of the exceptions, there were 9 (19%), 2 (4%), 8 (17%) and 3 (6%) cases of liver abscess, asymptomatic perihepatic fluid, pleural effusion and subcapsular hepatic hemorrhage, respectively, under CT guidance and 3 (6%), 1 (2%), 1 (2%) and 1 (2%) cases, respectively, under MR guidance. Of note, there were differences in the incidence of liver abscess (*P* = 0.022) and pleural effusion (*P* = 0.011) between the two guidance methods. Second, patients with subhepatic hemorrhage need to undergo interventional embolization, and patients with pleural effusion need to be treated with auxiliary thoracic drainage; these complications will undoubtedly prolong the patient’s hospital stay. None of the patients experienced life-threatening complications during or after treatment (Table 4**)**.

**Table 4:** Adverse events and complications

Categories	CT-guided (***n*** = 47)		MR-guided (***n*** = 54)		***P Value***
	Grades				
**Adverse events**	**CTCAE**		**CTCAE**		
Fever, maximum 38 °C, no treatment	I	17(36)	I	23(43)	0.546^*^
Fever, > 38 °C, treatment	II	13(28)	II	21(39)	0.293^*^
Nausea or vomiting	II	7(15)	II	6(11)	0.767^*^
Mild pain, requiring nonopioid oral analgesic treatment	II	24(51)	II	31(57)	0.553^*^
Moderate pain, requiring opioid oral analgesic treatment	II	18(38)	II	21(39)	1.000^*^
Mild liver dysfunction, requiring conservative treatment	II	32(68)	II	43(80)	0.254^*^
**complications**					
Liver abscess	III	9(19)	III	3(6)	0.022^*^
Asymptomatic perihepatic fluid	IV	2(4)	IV	1(2)	0.596^§^
Pleural effusion	III	8(17)	III	1(2)	0.011^*^
Subcapsular liver hemorrhage	IV	3(6)	IV	1(2)	0.336^§^

National Cancer Institute Common Terminology Criteria for Adverse Events (CTCAE version 4.03)

Data are numbers of events. Data in parentheses are percentages

Note. Data are presented as numbers of events. Data in parentheses are percentages. *Pearson χ2 test was used. §Fisher exact test was used

### Survival between two groups

**Fig. 5:**
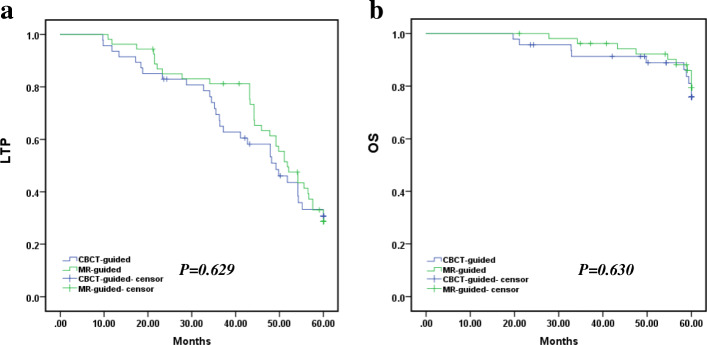
Kaplan–Meier local tumor progression (LTP) in the CT-guided group versus MR-guided group; **a.** Mean LTP was 44.264 months (95% CI: 39.484, 49.043) in the CT-guided group versus 47.745 months (95% CI: 43.840, 51.650) in the MR-guided group (*P* = 0.629, log-rank test). Kaplan–Meier overall survival (OS) of the CT-guided group versus the MR-guided group. **b.** The mean OS was 56.772 months (95% CI: 53.858, 59.889) in the CT-guided group versus 58.123 months (95% CI: 56.375, 59.889) in the MR-guided group (*P* = 0.630, log-rank test). The 1-, 3-, and 5-year LTP rates in patients in the CT-guided group were 93.6, 69.5 and 30.7%, respectively, and the 1-, 3- and 5-year OS rates were 100.0, 91.3 and 75.8%, respectively. The 1-, 3-, and 5-year LTP rates in the MR-guided group were 96.3, 81.2% and 28,7%, respectively, and the 1-, 3- and 5-year OS rates were 100.0, 96.2 and 79.4%, respectively

Next, Kaplan–Meier curves, local tumor progression (LTP) and overall survival (OS) were compared between the CT-guided group and the MR-guided group. The mean LTP was 44.264 months (95% CI: 39.484, 49.043) in the CT-guided group versus 47.745 months (95% CI: 43.840, 51.650) in the MR-guided group (*P* = 0.629, log-rank test). The mean OS was 56.772 months (95% CI: 53.858, 59.889) in the patients who underwent CT-guided procedures versus 58.123 months (95% CI: 56.375, 59.889) in those who underwent MR-guided procedures (*P* = 0.630, log-rank test). The 1-, 3-, and 5-year LTP rates of patients who underwent CT-guided procedures were 93.6, 69.5 and 30.7%, respectively, and the 1-, 3- and 5-year OS rates were 100.0, 91.3 and 75.8%, respectively. The 1-, 3-, and 5-year LTP rates of patients who underwent MR-guided procedures were 96.3, 81.2% and 28,7%, respectively, and the 1-, 3- and 5-year OS rates were 100.0, 96.2 and 79.4%, respectively **(**Fig. 5**)**.

### Factors affecting OS and LTP

Univariate Cox proportional hazard regression indicated that MWA under CT guidance versus MR guidance was not associated with longer LTP or OS (*P* = 0.632 and *P* = 0.633, respectively), while tumor diameter (≥3, < 5) (both *P* < 0.05), tumor location (challenging locations) (both *P* < 0.005) and the number of lesions (2–3 lesions) (both *P* < 0.001) were all related to shorter LTP and OS **(**Table 5**)**. Multivariate Cox regression also revealed that the guidance method used did not have a significant impact on LTP or OS and further showed that the tumor diameter (< 3 cm) and the number of lesions (single) could independently predict better LTP and OS (both *P* < 0.05).

**Table 5:** Factors affecting LTP and OS

Parameters	LTP			***P***	OS	*95%****CI***		***P***
	HR	*95%*CI			***HR***			
		Lower Higher				*Lower Higher*		
**Univariate Cox regression**								
Age (> 60 vs ≤ 60)	*0.217*	0.836	2.197	1.356	*2.825*	*1.085*	*7.356*	*0.033*
AFP (> 200 vs ≤ 200 ng/mL)	0.138	0.887	2.371	1.451	*1.496*	*0.619*	*3.613*	*0.371*
Tumor diameter (≥3,< 5 vs < 3 cm)	*2.644*	1.596	4.380	**0.000**	*2.865*	*1.100*	*7.460*	***0.031***
Tumor location (challenging location vs typical location)	*3.399*	2.065	5.593	**0.000**	*4.604*	*1.759*	*12.050*	***0.002***
Number of lesion (single vs 2–3 lesions)	*3.282*	1.995	5.399	**0.000**	*9.109*	*3.029*	*27.395*	***0.000***
Child–Pugh stage (B vs A)	*1.180*	0.723	1.923	0.508	*1.337*	*0.554*	*3.228*	*0.519*
Guidance system (CT vs MR)	*0.888*	0.547	1.443	0.632	*0.808*	*0.336*	*1.941*	*0.633*
**Multivariate Cox regression**								
Age (> 60 vs ≤ 60)	1.006	0.606	1.672	0.980	2.337	0.846	6.454	0.101
AFP (> 200 vs ≤ 200 ng/mL)	1.312	0.775	2.220	0.312	0.971	0.387	2.434	0.950
Tumor diameter (≥3,< 5 vs < 3 cm)	2.869	1.621	5.081	**0.000**	3.388	1.100	10.431	**0.033**
Tumor location (challenging location vs typical locations)	2.848	1.621	5.273	**0.001**	2.646	0.868	8.070	0.087
Number of lesion (single vs 2–3 lesions)	1.890	1.053	3.391	**0.033**	9.287	2.649	32.567	**0.000**
Child–Pugh stage (B vs A)	1.429	0.816	2.505	0.212	2.393	0.843	6.789	0.111
Guidance system (CT vs MR)	0.824	0.487	1.393	0.470	0.419	0.143	1.223	0.101

Note—general anesthesia (GA) and local anesthesia (LA); challenging locations (hepatic dome, close to the heart/diaphragm/hepatic hilum)

## Discussion

In the field of therapeutics, there are three main curative treatment options for early-stage HCC: hepatectomy, liver transplantation, or percutaneous ablation. Each method has limitations that need to be partially overcome to provide curative treatment for the largest number of patients and to avoid premature administration of palliative treatment for HCC. According to the guidelines of the EASL and the AASLD [10–12], local thermal ablation has been considered to be the first-line treatment option for patients with small HCC when the patient has comorbidities, liver dysfunction or limited surgical options. Percutaneous ablation involves a variety of techniques that have advanced over the last 10 years, enabling the treatment of an increasing number of patients with improved efficacy in terms of local control. Of note, RFA and MWA are the most commonly used thermal ablation methods for hepatic malignancies [13–15]. In comparison with RFA, MWA is a new method that offers similar benefits, such as a larger volume of cellular necrosis, reduction in the procedure time, and the ability to bring the target lesion to a higher temperature in a shorter period of time, and it is less susceptible to variation in the morphology of the treatment zone because of heat-sink effects from adjacent vasculature [16]. Additionally, a matching analysis of the propensity score between hepatic resection (HRN) and MWA therapy for single HCC ≤5 cm confirmed that the 5-year and 10-year OS rates of HRN were 76 and 47%, respectively, and the corresponding OS rates of MWA were 77 and 48% (*P* = 0.865) [17]. Another meta-analysis revealed that MWA may be superior to HRN, as it is as effective as HRN in terms of overall survival, disease-free survival, and tumor recurrence and is associated with fewer complications [18]. Therefore, MWA is highly valuable for the treatment of HCC.

Rempp et al. observed that all tumor progression occurs at the edge of the ablation zone [19], and previous research has repeatedly highlighted the importance of the safety margin in tumor ablation [20–22]. Although the mean safety margin based on the measured tumor diameter and ablation zone seemed to be sufficient, insufficient focal margins were detected in various cases, which may be the cause of local progression. Worth noting, although CT can meet the treatment requirements of MWA and provide accurate imaging, differentiation between vital tumor tissue and the ablation zone is possible only for a limited time after application of a contrast agent. Recently, MR-guided MWA has become commonly used as a minimally invasive therapy for the treatment of liver malignancies, which can clearly reveal the boundary between the burn range and normal tissue without the use of contrast agents [19, 23–25].

Weiss et al. [26] performed MWA on 50 patients under 1.5-T MR guidance and found that the median OS was 41.6 months, and only 4 patients (8%) had local recurrence after the procedure. Yang et al. [27] enrolled 26 patients (38 lesions) in their study, and the primary efficacy rate of MWA was 100%. It is strongly believed that magnetic resonance data have the strong advantage of allowing tumor assessment through 3D imaging, which can provide more details for reference during tumor treatment. In fact, MR-guided MWA allows successfully treat hepatic malignancies safely and with high technical efficacy, but the procedure durations are relatively long.

Currently, controlled studies on MR-guided MWA with HCC are extremely rare. Clasen et al. retrospectively compared the technical effects of CT-guided and MR-guided RFA for the treatment of HCC and found that these two guidance methods are both locally effective, and their research further revealed that MR-guided RFA may reduce the number of required sessions for complete tumor treatment [28]. A number of studies have shown that MWA and RFA have no effect on LTP and OS in HCC, and the number of adverse events are similar between the two methods [29–31]. In this research, univariate Cox proportional hazard regression indicated that MWA under CT guidance versus MR guidance had no correlation with longer LTP and OS (*P* = 0.632 and *P* = 0.633, respectively). However, tumor diameter (≥3 cm, < 5 cm), tumor location (challenging locations) and the number of lesions (2–3 lesions) were all related to shorter LTP and OS (both *P* < 0.05). Multivariate Cox regression further revealed that MR-guided MWA had no effect on LTP or OS (both *P* < 0.05), but the incidence of complications in the MR-guided group was lower (*P* < 0.05). Additionally, the duration of MR-guided MWA was shorter than that of CT-guided treatment, which may be related to the high-intensity resolution of MR in the ablation range. In fact, the more obvious signal changes around the tissue after thermal ablation could provide more accurate information for interventional therapy and indirectly shorten the ablation time.

There are several limitations to our research that should be noted. First, the real-time MR thermometry technique was not used in this study due to software limitations. In addition, the duration of MR-guided MWA is relatively longer than that of conventional CT-guided treatment. However, we have already used more optimized sequences to reduce the duration of the procedures and improve treatment efficiency. Finally, this is a single-center retrospective study involving a small number of cases, which may have led to biased results. Thus, further prospective studies and multicenter studies are needed, and the follow-up period should be extended to reduce the risk of bias.

## Conclusion

In summary, we have shown that both CT-guided and MR-guided MWA can safely and successfully treat HCC (≤5.0 cm) with high technical efficiency, but MR-guided MWA could reduce the occurrence of complications.

## Data Availability

The datasets generated for this study are available on request to the corresponding author.

## Abbreviations

MR: Magnetic resonance
CT: Computed tomography
MWA: Microwave ablation
HCC: Hepatocellular carcinoma
EASL: European Association for the Study of the Liver
AASLD: American Association for the Study of Liver Diseases
LTP: A local tumor progression
OS: Overall survival
RFA: Radiofrequency ablation
CA: Cryoablation
AFP: Alpha-fetoprotein
HRN: Hepatic resection
